# Liver Abnormalities in Systemic Lupus Erythematosus: A Prospective Observational Study

**DOI:** 10.7759/cureus.15691

**Published:** 2021-06-16

**Authors:** Shaik Imran, Molly Mary Thabah, Mohamed Azharudeen, Ananthakrishnan Ramesh, Zachariah Bobby, Vir S Negi

**Affiliations:** 1 Medicine, Jawaharlal Institute of Postgraduate Medical Education and Research (JIPMER), Puducherry, IND; 2 Radiodiagnosis, Jawaharlal Institute of Postgraduate Medical Education and Research (JIPMER), Puducherry, IND; 3 Biochemistry, Jawaharlal Institute of Postgraduate Medical Education and Research (JIPMER), Puducherry, IND; 4 Clinical Immunology, Jawaharlal Institute of Postgraduate Medical Education and Research (JIPMER), Puducherry, IND

**Keywords:** fatty liver, lupus hepatitis, transaminitis, sle, liver abnormalities, hepatic manifestations, drug induced hepatitis

## Abstract

Objectives

In this study, we aimed to examine and analyze liver abnormalities among patients with systemic lupus erythematosus (SLE), including both newly diagnosed patients and those being followed up, as well as the prevalence of lupus hepatitis.

Methods

This was a prospective observational study. Clinical data, liver function tests (LFTs), and the findings from the ultrasonography of the abdomen among the patients were prospectively recorded and evaluated.

Results

Overall, 28 of the total 135 (20.7%) patients had liver abnormalities, including biochemical and those detected via ultrasonography. Ten patients had transaminitis, defined as aspartate aminotransferase (AST) or alanine aminotransferase (ALT) levels >2 times the upper limit of normal (ULN). Nine patients had elevated alkaline phosphatase (ALP) or gamma-glutamyl transferase (GGT) of >2 times ULN. In three patients, transaminitis was due to anti-tubercular therapy (ATT)-induced hepatitis; in seven (5.2%), no specific cause for transaminitis could be identified, and hence they were classified as cases of lupus hepatitis. On comparing clinical features between patients with (n=7) and without lupus hepatitis (n=128), the condition was more prevalent in newly diagnosed SLE patients compared to those who had been on follow-up [six (85.7%) vs. 30 (23.6%), p=0.002]. All seven patients with lupus hepatitis had complete resolution of the transaminitis on follow-ups. However, one patient who had received ATT (isoniazid, rifampicin, ethambutol, and pyrazinamide) died. Ultrasonography showed fatty liver in seven patients and chronic liver disease in one patient.

Conclusion

In this study, transaminitis due to lupus hepatitis was seen in newly diagnosed lupus patients and was not associated with disease activity. Before diagnosing lupus hepatitis, drug-induced liver disease has to be ruled out, and if persistent LFT abnormalities are present, further workup is suggested to rule out overlap with primary biliary cirrhosis and/or autoimmune hepatitis.

## Introduction

Liver involvement in systemic lupus erythematosus (SLE) often manifests as abnormal liver enzymes [1,2]. The causes of liver function abnormalities in lupus are often secondary to drug toxicity, comorbidities like fatty liver, as well as chronic hepatitis B virus (HBV) and hepatitis C virus (HCV) infections [3-5]. Involvement of the liver in lupus can be due to overlap with autoimmune disorders such as autoimmune hepatitis and primary biliary cirrhosis [6], or it could be due to lupus disease itself (lupus hepatitis) [7,8].

The prevalence of lupus hepatitis in SLE patients has been reported to be around 3-23% [2,4,7,9], with some association with anti-ribosomal P antibodies [7]. The liver histology of lupus hepatitis is non-specific [10], and it is critical to rule out other causes of liver diseases including drug-induced liver injury, alcohol-related liver disease, and viral hepatitis before attributing the condition to lupus.

Practically, we often encounter patients with liver enzyme elevation, but such findings are overshadowed by more life-threatening renal and/or central nervous system involvement. Moreover, data on liver biochemical and clinical abnormalities among Indian lupus patients are scarce. In light of this, we performed an observational study to look at the frequency and etiology of liver abnormalities, especially the prevalence of lupus hepatitis.

## Materials and methods

This was a prospective observational study carried out from May 2018 to August 2019. Patients with SLE (both newly diagnosed and those under follow-up) aged ≥13 years who fulfilled the Systemic Lupus International Collaborating Clinics/American College of Rheumatology (SLICC/ACR) 2012 classification criteria [11] were eligible to be recruited after informed consent was obtained. The diagnosis was confirmed by the treating rheumatologist. Those patients with overlap syndromes were excluded. Baseline demographic characteristics and lupus clinical manifestations such as mucocutaneous, musculoskeletal manifestations, thrombocytopenia (platelet count <100,000/mm^3^), leucopenia (total leukocyte count <4,000/mm^3^), autoimmune hemolytic anemia, lupus nephritis, neuropsychiatric SLE (NPSLE), serositis, and pericardial effusion were noted. Disease activity was assessed according to the Systemic Lupus Erythematosus Disease Activity Index 2000 (SLEDAI-2K) [12].

History of liver and gastrointestinal symptoms, namely jaundice, weight loss, fatigue, pruritus, and pain in the abdomen, was taken. Clinical examination was performed to look for the presence of jaundice, hepatomegaly, splenomegaly, and ascites. Medication intake, specifically any known hepatotoxic drugs, was noted. Human immunodeficiency virus (HIV), hepatitis B surface antigen (HBsAg), and HCV serology were tested in all patients. Liver function tests (LFTs) included serum bilirubin, total protein, albumin, globulins, alanine aminotransferase (ALT), aspartate aminotransferase (AST), alkaline phosphatase (ALP), and gamma-glutamyl transferase (GGT).

Ultrasonography of the abdomen for liver echotexture and presence of portal hypertension and/or other structural abnormalities was performed using Esaote MyLab™ ultrasound machine (Esaote SpA, Genoa, Italy) with a convex transducer of a frequency of ~5 MHz. Fatty liver was graded as follows - grade 1: diffusely increased hepatic echogenicity, but periportal and diaphragmatic echogenicity was still appreciable; grade 2: diffusely increased hepatic echogenicity obscuring periportal but not diaphragmatic echogenicity; and grade 3: diffusely increased hepatic echogenicity obscuring periportal as well as diaphragmatic echogenicity [13].

An abnormal LFT was defined as an increase of ≥2 times the upper limit of normal (ULN) in either AST (normal range: 0-40 IU/L) or ALT (normal range: 0-45 IU/L) or ALP (normal range: 30-125 IU/L) or GGT (normal range: 1-50 IU/L). Transaminitis was defined as an increase of ≥2 times ULN in either AST or ALT. Follow-up LFTs at discharge or at two weeks were checked for those patients who had any abnormality to see if they had resolved or worsened. Liver abnormality was defined as the presence of abnormal findings either on LFT or abdominal ultrasonography.

Statistical analysis

The estimated minimum sample size was 93, based on an expected prevalence of liver abnormalities among SLE patients of 40%, using the statistical formula for estimating a single proportion, at a 5% level of significance and 25% relative precision. Data were analyzed using the SPSS Statistics software version 21 (IBM, Armonk, NY). Comparison of categorical variables between those with and without lupus hepatitis was done by Chi-square test or Fisher’s exact test as appropriate. Statistical analysis was carried out at a 5% level of significance and a p-value <0.05 was considered significant. All patients provided written informed consent, and the study protocol was approved by the Jawaharlal Institute of Postgraduate Medical Education and Research Institutional Ethics Committee (Human Studies) vide letter no. IEC Ref. JIP/IEC/2017/0401.

## Results

The study involved a total of 135 SLE patients; the median age of the patients was 28 years. Five patients were aged less than 18 years. Thirty-six (26.6%) of the 135 patients were new admissions in whom SLE had been diagnosed for the first time. The median SLEDAI-2K score was 13 (IQR: 4-28). Sixty-eight (50.3%) patients had high disease activity (SLEDAI-2K: >10), and 67 (49.7%) had low to moderate disease activity (SLEDAI-2K: ≤10). Baseline characteristics of the cohort are summarized in Table 1.

**Table 1:** Baseline characteristics of patients with SLE *Values expressed are numbers and percentages, unless otherwise indicated IQR: interquartile range; SLE: systemic lupus erythematosus; SLEDAI-2K: Systemic Lupus Erythematosus Disease Activity Index 2000

Variables*	Values (n=135)
Age in years, median (IQR)	28 (15-57)
Women	130 (96)
Duration of SLE in months, median (IQR)	12 (3-30)
SLEDAI-2K score, median (IQR)	13 (4-28)
Malar rash	86 (63.7)
Alopecia	100 (74.1)
Oral ulcers	103 (76.3)
Arthritis/arthralgia	91 (67.4)
Serositis (either pleural or pericardial effusion)	8 (5.9)
Myositis	2 (1.5)
Pleural effusion/pleuritis	2 (1.48)
Pericardial effusion	6 (4.44)
Neuropsychiatric manifestations	15 (11.1)
Vasculitis	25 (18.5)
Lupus nephritis	66 (48.9)
Antiphospholipid syndrome	8 (5.92)
Hematological involvement	65 (48.1)
Leukopenia (total leukocyte count <4,000/mm^3^)	6 (4.4)
Thrombocytopenia (platelets <100,000/mm^3^)	18 (13.3)
Positive direct Coombs test	9 (6.6)
Anemia (hemoglobin <10 g/dL)	55 (40.7)
Extractable nuclear antigens (ENA)	
Anti-nucleosome	18 (16.6)
Anti-histone	18 (16.6)
Anti-U1RNP	20 (19)
Anti-Sm	30 (27)
Anti-ribosomal P	4 (2.3)
Anti-ds DNA	45 (55)
Anti-mitochondrial antibodies	2 (1.2)
Anti-Ku	5 (3.5)
Anti-SSA	22 (23.8)
Anti-SSB	7 (6)

Lupus nephritis (biopsy-proven) was present in 66 (48.9%) patients with class IV lupus nephritis as the most common subtype. Among those patients who were on follow-up, 72% were on steroids. Sixty-four of the follow-up patients had received immunosuppressive treatment with either intravenous cyclophosphamide or azathioprine or mycophenolate mofetil. All patients were on hydroxychloroquine. None of the patients were on long-term non-steroidal anti-inflammatory drugs (NSAIDs) or statins or any known hepatotoxic drugs (such as leflunomide) at the time of enrolment. There was no history of habitual consumption of alcohol in any of the patients. All patients were negative for HBsAg, HIV, and anti-HCV antibodies.

None of the patients had a history of symptoms to suggest autoimmune hepatitis or primary biliary cirrhosis, such as pruritus, weight loss, fatigue, or significant abdominal pain. On clinical examination, jaundice was present in two patients, mild hepatomegaly in two patients, and mild splenomegaly in three patients. Of the two patients with hepatomegaly, one had Hodgkin’s lymphoma and the other had fatty liver on ultrasonography. Of the three patients with clinically mild splenomegaly, one patient had Hodgkin’s lymphoma (mentioned earlier), the second patient had splenic abscesses and succumbed to sepsis, and the third patient had portal hypertension and chronic liver disease as revealed on ultrasonography. This patient also succumbed to pneumonia and sepsis. However, a liver biopsy could not be performed, and the differential diagnosis that was considered was non-alcoholic steatohepatitis.

LFTs showed that 10 patients had transaminitis and six patients had hyperbilirubinemia. In three patients, transaminitis was drug-induced: two had pulmonary TB and were on anti-tubercular therapy (ATT), and their transaminitis was attributed to ATT-induced hepatitis; the third patient had pneumonia and sepsis; he was on empirical ATT and had developed ATT-induced hepatitis, and finally succumbed. This particular patient also had a prolongation of prothrombin time (PT) with an international normalized ratio (INR) >5.5; in the other two patients, PT was normal. All these three patients had received a combination of isoniazid, rifampicin, ethambutol, and pyrazinamide.

In seven patients (5.2%), no other cause for transaminitis could be identified, and hence the transaminitis was classified as lupus hepatitis. Six of these patients with lupus hepatitis were newly diagnosed, and none of them were on azathioprine, which is a potential hepatotoxic drug. The remaining one patient had defaulted treatment and presented with a renal flare and was found to have transaminitis. The mean AST and ALT in these seven patients were 154 mg/dl and 80 mg/dl respectively. None of these patients had either subclinical or clinical myositis. On follow-up, LFTs showed complete resolution of the transaminitis, and a liver biopsy was not indicated. Clinical features of patients having lupus hepatitis (n=7) vs. those without lupus hepatitis (n=128) are compared in Table 2. 

**Table 2:** Comparison of clinical and laboratory features between those with and without lupus hepatitis SLEDAI-2K: Systemic Lupus Erythematosus Disease Activity Index 2000; low C3: low serum complement C3 (normal range: 0.9-1.8 g/L); low C4: low serum complement C4 (normal range: 0.1-0.4 g/L)

Parameter	Lupus hepatitis, n=7, n (%)	No lupus hepatitis, n=128, n (%)	P-value	OR (95% CI)
Female	7 (100)	123 (94.6)	1	0.9 (0.9-1)
Newly diagnosed	6 (85.7)	30 (23.6)	0.002	19.4 (2.2-167)
SLEDAI-2K score ≥10	3 (43)	64 (50)	1	0.75 (0.16-3.9)
Prednisolone use	6 (85.7)	67 (52.3)	0.12	5.4 (0.64-46)
Arthritis	5 (71.4)	86 (67.2)	1	0.9 (0.9-1)
Myositis	0	2 (1.6)	1	1 (1.01-1.09)
Malar rash	4 (57)	82 (64)	0.7	0.7 (0.16-3.5)
Alopecia	7 (100)	93 (73)	0.19	0.9 (0.8-1)
Oral ulcers	6 (87.5)	97 (76)	0.19	0.9 (0.8-1)
Vasculitis	1 (14.3)	24 (19)	1	0.7 (0.08-6.2)
Neuropsychiatric manifestations	0	15 (11.7)	1	1.06 (1.06-1.1)
Lupus nephritis	3 (43)	63 (49)	1	0.7 (0.16-3.5)
Serositis	1 (14.3)	7 (5.5)	0.3	2.8 (0.3-27)
Hematological involvement	4 (57)	61 (47.7)	0.7	1.4 (0.3-6.8)
Platelet count <100,000/mm^3^	2 (28.6)	16 (12.5)	0.23	2.8 (0.5-15.7)
Hemoglobin <10 g/dL	4 (57)	51 (40)	0.4	2 (0.4-9.3)
Low C3	4 (57)	60 (47)	0.7	1.5 (0.3-7)
Low C4	3 (43)	45 (35)	0.7	1.3 (0.3-6.4)
Serum albumin <3.5 g/dL	5 (71.5)	52 (40.6)	0.13	3.6 (0.7-19.5)
Hepatomegaly	0	2 (1.6)	0.7	1.05 (1.01-1.1)
Splenomegaly	0	3 (2.3)	1	1.05 (1.01-1.1)
Fatty liver	0	6 (4.7)	1	1 (1.01-1.1)

Six (85.7%) newly diagnosed patients had lupus hepatitis compared to only 30 (23.6%) patients who were on follow-up (p=0.002). There were no differences in other clinical features or SLEDAI-2K scores between those with or without lupus hepatitis. Antibodies to extractable nuclear antigens (ENA) by line immunoassay were available for 84 patients (Table 1). Among these, anti-ribosomal P was positive in only four (2.3%) patients, and anti-mitochondrial antibodies were positive in two (1.2%). None of the patients with lupus hepatitis was positive for anti-ribosomal P.

On ultrasonography of the abdomen, nine patients had abnormal findings: altered liver echotexture with portal hypertension (n=1), hepatomegaly due to Hodgkin’s lymphoma (n=1), and fatty liver (n=7). Of the seven patients with fatty liver, four had grade 1, and three had grade 2 fatty liver. None of the patients with fatty liver had liver enzyme abnormalities.

As expected, there were overlaps in LFT findings, as summarized in Table 3; there were 19 patients with any one enzyme elevation. Summing up with abnormal ultrasonography findings of nine patients, the total number of patients with liver abnormalities was 28 out of 135 (20.7%).

**Table 3:** Liver function test findings in patients with systemic lupus erythematosus (n=135) AST: aspartate aminotransferase; ALT: alanine aminotransferase; ALP: alkaline phosphatase; GGT: gamma-glutamyl transferase; ULN: upper limit of normal

Parameters	N (%)
AST >2 times ULN	10 (7.4)
ALT >2 times ULN	6 (4.4)
Transaminitis (AST or ALT >2 times ULN)	10 (7.4)
Hyperbilirubinemia (serum bilirubin >1.2 mg/dL)	6 (4.4)
ALP >2 times ULN	4 (2.9)
GGT >2 times ULN	11 (8.14)
Hypoalbuminemia (serum albumin <3.5 g/dL)	57 (42.2)
Hypergammaglobulinemia (serum globulin >3.5 g/dL)	59 (43.4)
Prolonged prothrombin time (PT)	1 (patient had sepsis)

## Discussion

In this study, seven (5.2%) patients had transaminitis that could not be attributed to anything other than lupus alone, and hence they were classified as cases of lupus hepatitis [2,7]. The prevalence of lupus hepatitis varies widely from 3 to 23% [1,2,4,7,14]. In one of the largest studies on lupus hepatitis, a 24-month prospective study on 260 patients, 8% of the patients were classified as cases of lupus hepatitis [2]. Arnett et al., in a retrospective analysis, found that 4/131 patients (3%) had lupus hepatitis [7]. In a more recent study, a retrospective analysis of 242 patients, 5.8% of patients had liver enzyme abnormalities that could only be attributed to SLE [14]. Thus, it appears that the prevalence of lupus hepatitis in our study (5.2%) is similar to these studies [2,7,14].

Studies that reported a higher percentage of overall liver abnormalities had a relatively larger number of patients who were on NSAIDs, aspirin, or methotrexate [14,15]. A Japanese study reported the presence of liver dysfunction in 123 of 206 (59%) patients [15]. Of those 123 cases, one-third was drug-induced, and another one-third was due to SLE; other causes were fatty liver, autoimmune hepatitis, etc. This study showed that liver dysfunction developed largely at the onset of SLE [15]. We also observed that transaminitis was more commonly present in newly diagnosed patients, and there was no association with disease activity. However, a few studies have found an association between lupus hepatitis and disease activity [2,8].

Of the various lupus-specific autoantibodies, anti-ribosomal P has been found to be prevalent in lupus hepatitis [7,16]. The other autoantibody sometimes associated with lupus hepatitis is the anti-smooth muscle antigen [9]. Anti-mitochondrial antibodies, a specific marker for primary biliary cirrhosis, can be found in some proportion of patients with lupus [17]. From a clinical perspective, the primary purpose of testing for autoantibodies in SLE hepatitis is to diagnose co-existing autoimmune hepatitis. Because, while lupus hepatitis is benign, autoimmune hepatitis often needs more intensive treatment.

It was observed in our study that there was a striking elevation of AST/ALT (>10 times) in those with drug-induced hepatitis, while the patients with lupus hepatitis had only mild elevations. None of our patients were on native or herbal medicines and none had chronic viral hepatitis. In contrast, a study from Korea has reported a significantly large number of patients having transaminitis due to herbal medication use [18]. Another observation we made was the relative elevation of AST over ALT in patients with lupus hepatitis. It has been suggested that higher levels of AST compared to ALT occur due to excessive hepatic oxidative stress [9].

Hypoalbuminemia, an important marker of inflammation, was significantly more prevalent among patients with transaminitis. Physiological levels of bilirubin and albumin represent major antioxidant components in the serum [19], and patients with active SLE often have subnormal levels of serum bilirubin [20].

The other significant liver abnormality seen in our study was the presence of fatty liver on ultrasonography of the abdomen. Fatty liver or non-alcoholic steatohepatitis is part of the spectrum of non-alcoholic fatty liver disease. The co-existence of symptomatic non-alcoholic steatohepatitis with SLE sometimes leads to liver failure [21]. Some studies have reported that fatty liver reverses after treatment with prednisolone; therefore, it has been speculated that fatty liver can be a direct manifestation of lupus [22,23]. 

The prevalence of asymptomatic fatty liver in lupus varies widely [3,4,24,25]. A case series from Japan has reported that 4.6% of patients have fatty liver [25], whereas a retrospective analysis of 134 Canadian lupus patients showed that 41% had fatty liver [4]. High prevalence of methotrexate and NSAIDs usage, long duration of SLE, accelerated atherosclerosis [4], and high-dose glucocorticoid use could explain the presence of fatty liver [24]. Fatty liver has also been reported in an adolescent with SLE, suggesting that lupus per se can cause fatty liver [26]. In contrast, none of our patients were on methotrexate or long-term NSAIDs. While lupus patients can have non-alcoholic steatohepatitis as comorbidity, on the other hand, about 21% of patients with non-alcoholic steatohepatitis can have moderate titers anti-nuclear antibodies (ANA) that are associated with more inflammation though not more advanced non-alcoholic steatohepatitis [27]. In an ideal setting, lupus patients with fatty liver merit a liver biopsy to confirm the diagnosis of non-alcoholic steatohepatitis.

The major drawback of our study was that a complete autoantibody profile, namely anti-ribosomal P and the liver panel autoantibodies, could not be performed. However, it could be said that our findings actually represent real-world information as ENA is not routinely required in day-to-day practice. A potential confounder to the true prevalence of lupus hepatitis was the inclusion of follow-up patients. If we had studied only newly diagnosed patients who had not been started on treatment, we would probably have been able to know the real prevalence of lupus hepatitis.

Liver biopsy was not indicated in any of our patients with lupus hepatitis, because the liver enzymes had returned to baseline on follow-up. Lupus hepatitis perhaps has no typical histopathological findings [10]. But a liver biopsy is useful to exclude associated liver pathologies [12]. The presence of positive complement C1q deposits in the liver is characteristic of lupus hepatitis.

## Conclusions

In our study, transaminitis due to lupus hepatitis was seen in newly diagnosed lupus patients and was not associated with disease activity. Before diagnosing lupus hepatitis, drug-induced liver disease has to be ruled out. In patients whose liver abnormalities persist, further workup is suggested to rule out overlap with autoimmune hepatitis or primary biliary cirrhosis. Moderate to severe transaminitis and hyperbilirubinemia are more often due to adverse events related to drugs. The risk factors and prognosis of fatty liver among lupus patients need to be studied further, especially since it has been observed that ANA can be present in a significant proportion of patients with non-alcoholic steatohepatitis.
